# ^19^F-NMR spectroscopy of fluorinated isoleucine analogues in a protein

**DOI:** 10.1007/s10858-026-00488-z

**Published:** 2026-02-17

**Authors:** Adarshi P. Welegedara, Yi Jiun Tan, Matteo Borgini, Peter Wipf, Gottfried Otting

**Affiliations:** 1 ARC Centre of Excellence for Innovations in Peptide & Protein Science, Research School of Chemistry, Australian National University, Canberra, ACT 2601 Australia; 2 Department of Chemistry, University of Pittsburgh, Pittsburgh, PA 15260 USA; 3 School of Pharmacy, University of Eastern Finland, Kuopio, 70210 Finland; 4 Department of Chemistry and Biochemistry, Augusta University, Augusta, GA 30912 USA

**Keywords:** CH_2_F group, CHF_2_ group, ^19^F-NMR spectroscopy, Fluoroisoleucine, Difluoroisoleucine, R3H domain

## Abstract

The R3H domain of the human protein Sµbp-2 was produced with 5-fluoro-L-isoleucine (FIle) and 5,5-difluoro-L-isoleucine (diFIle) as probes for detection by ^19^F-NMR spectroscopy. The fluorinated protein, produced by cell-free protein synthesis, was obtained more easily with diFIle than FIle as FIle readily hydrolysed at pH 7.5 with the release of fluoride. The ^19^F-NMR spectra showed large chemical shift ranges but were heterogeneous. The heterogeneities arose from difficulties to fully exclude canonical isoleucine, the presence of multiple conformations and limited stability of the proteins, with the sample made with diFIle being particularly prone to precipitation. ^19^F resonance assignments were obtained by comparison of the chemical shifts of γ_1_-protons with those observed in the wild-type protein. Non-uniform cross-peak intensities observed in short-delay ^1^H,^19^F correlation experiments suggest incomplete averaging of ^3^*J*_HF_ couplings and therefore preferential rotamer populations of the CH_2_F and CHF_2_ groups.

**Supplementary Information:**

The online version contains supplementary material available at 10.1007/s10858-026-00488-z.

## Introduction

**Fig. 1:**
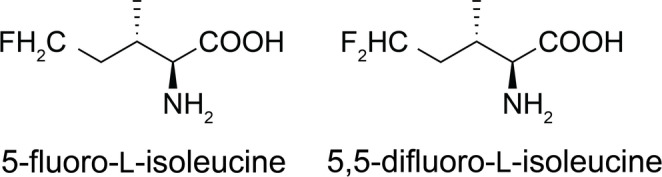
Chemical structures of 5-fluoro-L-isoleucine (FIle) and 5,5-difluoro-L-isoleucine (diFIle)

Proteins produced with global high-level substitution of valine and leucine by fluorinated analogues yield ^19^F-NMR spectra with large chemical shift dispersion. Fluorinated valine and leucine analogues tested include variants where a fluorine atom is installed in either one or both methyl groups to yield CH_2_F groups (Tan et al. 2024, 2025; Frkic et al. 2024a, b; Abdelkader et al. 2025). The chemical synthesis of these amino acids with stereoselective installation of fluorine in methyl groups has been described (Maleckis et al. 2022) and commercial suppliers (f. ex. Enamine) have worked to make them available. Although the aminoacyl-tRNA synthetases involved in the biosynthesis of proteins prefer canonical amino acids, they also accept fluorinated analogues, and proteins with high levels of fluorinated amino acids can be prepared by cell-free protein synthesis conducted without the corresponding canonical amino acid. The ^19^F-NMR signals of buried residues are significantly broader than those of solvent-exposed CH_2_F groups and in work conducted with the B1 domain of protein G (GB1), the signals of buried difluorovaline residues were broader than those of buried difluoroleucine residues (Tan et al. 2025; Abdelkader et al. 2025), suggesting that greater flexibility of the CH_2_F groups fosters narrower signals. This hypothesis prompted us to investigate the performance of fluorinated isoleucine analogues with fluorine on the δ-carbon. Figure 1 shows the isoleucine analogues tested.

As GB1 contains only a single isoleucine residue which is also highly solvent-exposed, we used the R3H domain of the human protein immunoglobulin S-µ-binding protein 2 (Sµbp-2) to probe the performance of FIle and diFIle. This R3H domain has a molecular weight of about 9.5 kDa and contains three isoleucine residues (Ile733, Ile761 and Ile780). Their sidechains participate in the hydrophobic core of the domain. The NMR structures of the R3H domain (PDB IDs 1MSZ and 2LRR) show that the C^δ^ atoms of Ile733 and Ile761 are within 3.8 − 5.1 Å of each other (Liepinsh et al. 2003; Jaudzems et al. 2012), suggesting that δ-fluorine atoms in these groups could make transient fluorine−fluorine contacts detectable in [^19^F,^19^F]-TOCSY spectra (Orton et al. 2021; Tan et al. 2025; Abdelkader et al. 2025).

In the following we describe the production of the R3H domain with FIle (R3H-F) and diFIle (R3H-F2) using cell-free protein synthesis, the ^19^F-NMR spectra obtained, and resonance assignments made.

## Results

### Protein preparations

The proteins were produced by cell-free protein synthesis (CFPS) using standard protocols (Apponyi et al. 2008; Ozawa et al. 2012; Wu et al. 2007; details provided in the Supporting Information). FIle·HCl was purchased from Enamine (Ukraine) but only 5 mg of diFIle·HCl were available from a synthesis program concluded 3 years ago (Borgini and Wipf 2022). A 1D ^19^F-NMR spectrum recorded of diFIle in aqueous solution showed two different species in 3:2 ratio (Figure S1), different from the single ^19^F-NMR signal in DMSO-d_6_ reported previously. Relying on the stereoselectivity of the isoleucyl-tRNA synthetase and the peptidyl-transfer centre of the ribosome, we used the complete amino acid material to produce a single sample of R3H-F2 in 1 mL inner reaction mixture of the CFPS setup. To maximise the protein yield, cleavage of the N-terminal His_6_-tag was omitted. Following purification on a Ni-NTA column, 2.3 mg of protein were obtained. For comparison, about 4 mg of wild-type R3H domain were obtained under the same CFPS conditions with provision of 1 mM canonical isoleucine. Intact protein mass spectrometry showed that protein containing two diFIle residues and one canonical isoleucine was slightly more abundant than protein containing diFIle at all three sites (Figure S2b), indicating that, for each of the three sites, the statistical probability of finding diFIle was about 58%.

Producing the R3H domain made with FIle (R3H-F) was more difficult than the production of R3H-F2 because FIle proved to be unstable at the pH used for CFPS (pH 7.5). Incubation at 30 °C in 55 mM HEPES buffer resulted in the loss of three quarters of the ^19^F-NMR signal of the amino acid after one hour and the concomitant appearance of the signal of fluoride. Use of lower pH values seriously compromises CFPS yields and, in any case, conducting the control experiment at pH 7.0 only halved the rate of FIle degradation. FIle is known to be prone to forming 3-methylproline by intramolecular nucleophilic attack of the amino group on the CH_2_F group (Hudlický et al. 1970). Conducting the CFPS reaction for longer than 1 h without provision of fresh FIle resulted in protein that predominantly contained isoleucine instead of FIle. To maximize the exchange across the dialysis membrane separating the inner and outer reaction mixtures of the CFPS setup, the final R3H-F sample was produced in 40 separate batches of CFPS reactions with 1 mL inner buffer volume, conducting the reactions for only 6 h and providing fresh FIle from a 100 mM stock solution every hour. In this way, 4 mg of purified R3H-F were obtained. Intact protein mass spectrometry indicated that R3H-F contained FIle at any one of the three isoleucine sites with a probability of about 84% (Figure S2c).

## Comparison of the stabilities of FIle and corresponding fluoroleucine isomers

To compare the rate of hydrolysis of FIle with those of the previously used amino acids (2*S*,4*S*)-5-fluoroleucine (FLeu1) and (2*S*,4*R*)-5-fluoroleucine (FLeu2; Tan et al. 2024; 2025), we incubated the HCl salts of FLeu1 and FLeu2 under the same conditions (55 mM HEPES buffer pH 7.5, 30 °C), monitoring the heights of the fluorine signals and the appearance of inorganic fluoride. In these experiments, FLeu1 remained unaltered after 1 h and FLeu2 lost less than 10% of its ^19^F-NMR signal with the concomitant appearance of fluoride.

### 1D ^19^F-NMR

**Fig. 2:**
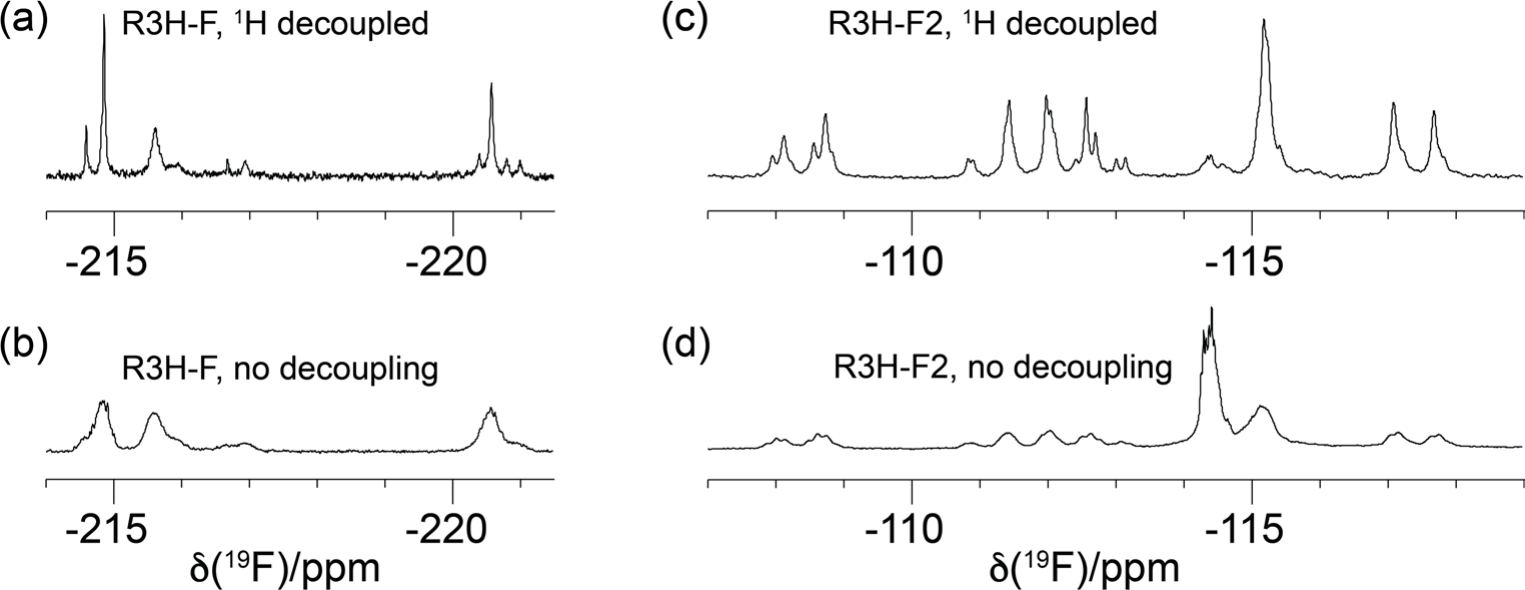
1D ^19^F-NMR spectra of R3H-F and R3H-F2 recorded at 25 °C. (a) Spectrum of R3H-F recorded with ^1^H decoupling, using 223 scans. The initial sample concentration was about 800 µM. (b) Same as (a), but without decoupling. 1024 scans. (c) Spectrum of R3H-F2 recorded with ^1^H decoupling. 2024 scans. The initial sample concentration was about 500 µM. (d) Same as (c) but recorded 4.5 days later without decoupling. 20,240 scans. Sample degradation was indicated by greatly decreased sensitivity and the new peak at -114.4 ppm

Unless mentioned otherwise, all NMR spectra were recorded on a 500 MHz NMR spectrometer equipped with a cryoprobe. Figure 2 shows the 1D ^19^F-NMR spectra of R3H-F and R3H-F2. The spectra recorded with ^1^H decoupling (Fig. 2a and c) show evidence of sample heterogeneities as expected, if the ^19^F chemical shifts are sensitive to the presence or absence of other fluorinated residues in the vicinity. Nonetheless, the different species detected in the spectrum of R3H-F2 (Fig. 2c) show very similar chemical shifts, suggesting that the different species differ more subtly than expected for the different diastereomers of the diFIle amino acid used to produce the protein (Figure S1) and vindicating the assumption that the isoleucyl-tRNA synthetase and the peptidyl-transfer centre of the ribosome select the diastereomer of canonical isoleucine.

Without decoupling, the spectrum of R3H-F shows three main peaks of similar size (Fig. 2b). The sample precipitated during the NMR measurements, reducing the signal intensity to a third over the course of 4.5 days. In the case of R3H-F2, the undecoupled spectrum (Fig. 2d) shows basically the same signals as the spectrum recorded with ^1^H decoupling (Fig. 2c) except for broader apparent line widths and the presence of a new signal at -114.4 ppm, which increased as the sample precipitated during NMR measurements. Only the large ^2^*J*_FF_ coupling was resolved in the spectra of R3H-F2, whereas the 2-bond and 3-bond ^1^H−^19^F couplings observed for the free amino acid (Figure S1) remained unresolved. After 4.5 days of NMR measurements, the signal intensity was reduced about five-fold due to sample degradation.

The line widths at half maximum were as narrow as 15 Hz for R3H-F (50 Hz for the broad signal at -215.6 ppm) with ^1^H decoupling (Fig. 2a). The line widths of R3H-F2 were about 30 Hz (Fig. 2c). A measurement of *T*_1ρ_(^19^F) using a CPMG train of 180^o^(^19^F) pulses spaced by 2 ms delays yielded values of 21 ms, 41 ms and 12 ms for residues 733, 761 and 780, respectively. Without ^1^H decoupling, the line widths at half maximum were about 140 Hz in R3H-F and 120 Hz in R3H-F2 (Fig. 2b and d). The broader signal envelope in R3H-F is expected for the presence of two large ^2^*J*_HF_ couplings rather than one. To probe the impact of the second fluorine in diFIle on longitudinal relaxation, we performed inversion-recovery experiments with R3H-F2, which yielded *T*_1_(^19^F) values ranging between 0.2 and 0.3 s (with residue 780 relaxing most rapidly and residue 733 slightly faster than residue 761). The sample heterogeneities and limited sample lifetimes made it challenging to record 2D NMR spectra.

### 2D NMR of R3H-F

**Fig. 3:**
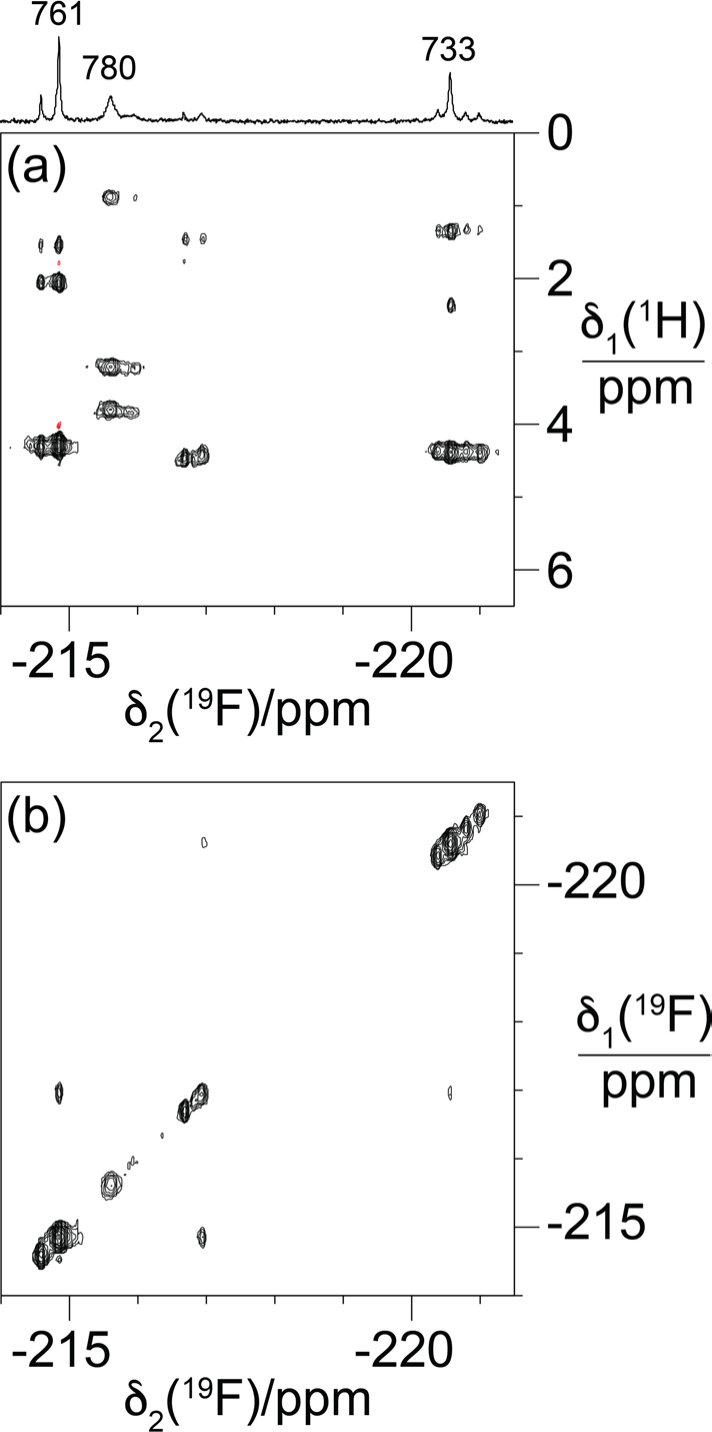
2D NMR spectra of R3H-F. (a) Short-delay ^1^H,^19^F correlation experiment (Tan et al. 2024). Parameters used: Δ_1_ = 7 ms, Δ_2_ = 2.5 ms, *t*_1max_ = 7 ms, *t*_2max_ = 102 ms, total recording time 2 h. The 1D ^19^F-NMR spectrum is plotted at the top with the resonance assignments. (b) [^19^F,^19^F]-TOCSY spectrum. Parameters used: 43 ms mixing time (DIPSI-2 mixing), *t*_1max_ = 5.4 ms, *t*_2max_ = 102 ms, total recording time 10.5 h

A NOESY spectrum of R3H-F confirmed the conservation of its three-dimensional structure by the conservation of ^1^H chemical shifts and NOE patterns. The ^19^F-NMR resonances were assigned by a short-delay ^1^H,^19^F correlation experiment (Tan et al. 2024), which yielded cross-peaks with the protons of the C^δ^H_2_F and C^γ1^H_2_ groups (Fig. 3a). The spectrum shows the ^1^H-NMR signals of the C^δ^HF_2_ groups between 3 and 4.5 ppm and of the C^γ1^H_2_ groups between 0.5 and 2.5 ppm. Comparison of the ^1^H chemical shifts observed in this spectrum with those of the wild-type R3H domain reveals conservation of the relative chemical shifts of the ^1^H resonances of the FIle side chains. For example, in the wild-type protein, the ^1^H-NMR signals of the C^γ1^H_2_ and C^δ^H_3_ groups of Ile780 are significantly shifted upfield by ring-currents from His759, while the ^1^H-NMR signals of Ile733 and Ile761 are closer to each other with those of Ile733 slightly more down-field. Furthermore, Ile733 features a larger difference in chemical shifts between its γ_1_-protons than Ile761. The same features are recapitulated in the spectrum of Fig. 3a.

The attempt to detect through-space scalar ^19^F−^19^F couplings in R3H-F between residues 733 and 761 by [^19^F,^19^F]-TOCSY spectra failed. Figure 3b shows that the only cross-peaks detected were with minor signals at about − 217 ppm. The most plausible interpretation of these cross-peaks is their origin in chemical exchange rather than scalar couplings, as a [^19^F,^19^F]-TOCSY spectrum recorded with 70 ms mixing time did not yield significantly increased cross-peaks as expected for the tan^2^(π*J*τ_m_) dependence of the intensity ratio of TOCSY cross-peaks over diagonal peaks, where τ_m_ is the mixing time (Braunschweiler and Ernst 1983). As cross-peaks associated with small through-space couplings feature with much greater intensity in TOCSY than NOESY spectra (Orton et al. 2021; Tan et al. 2024, 2025; Abdelkader et al. 2025), we also recorded a [^19^F,^19^F]-NOESY spectrum. This spectrum showed the same cross-peaks with similar intensities relative to the diagonal peaks (Figure S3). As the cross-peaks with residues 733 and 761 do not line up exactly, the respective exchange peaks appear to be with two different unidentified minor species with slightly different chemical shifts near − 217 ppm.

**Fig. 4:**
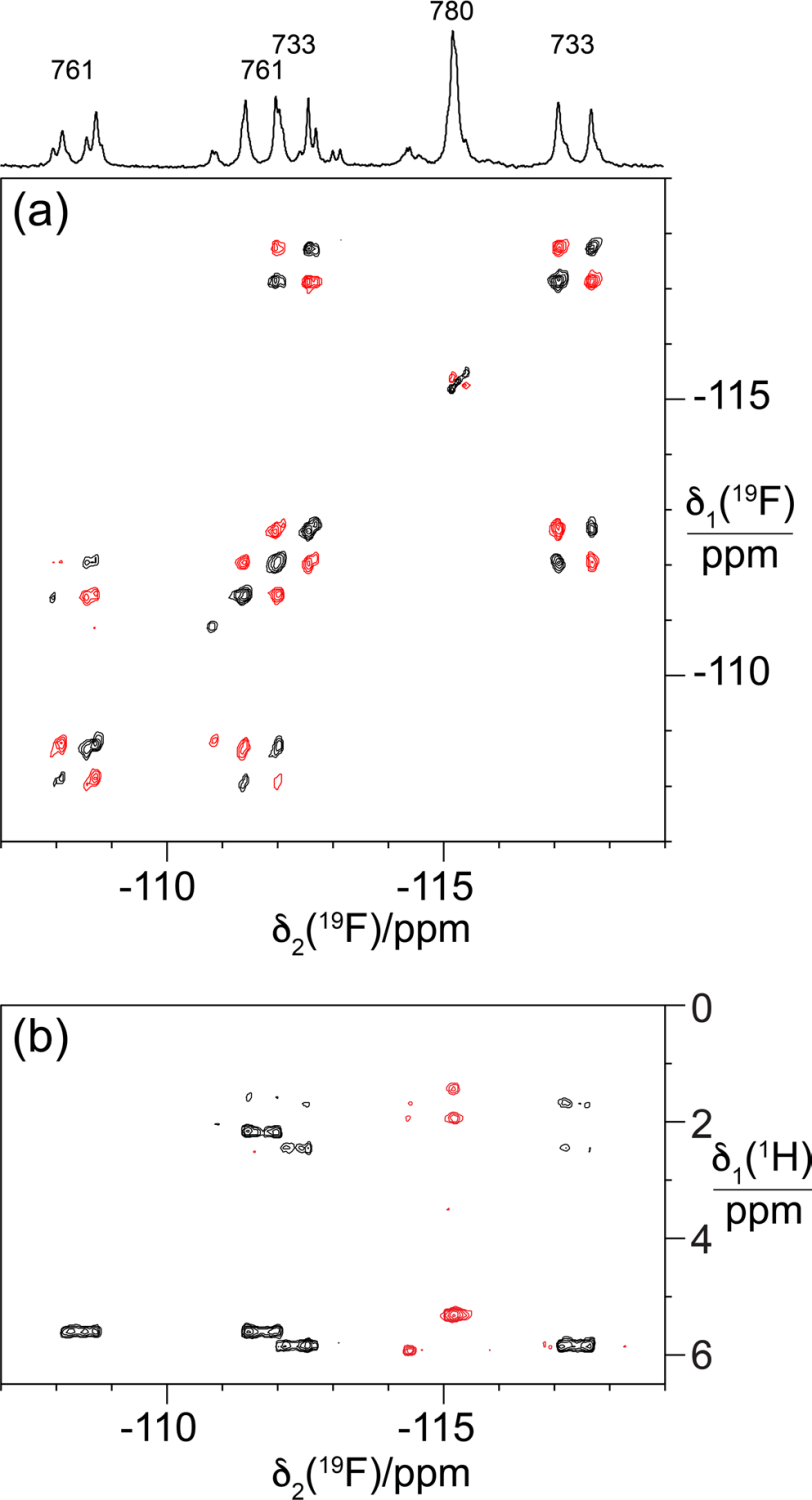
NMR spectra of R3H-F2. (a) ^19^F-^19^F DQF-COSY spectrum. The 1D ^19^F-NMR spectrum of Fig. 3c is plotted at the top with the resonance assignments. Parameters used: *t*_1max_ = 6.7 ms, *t*_2max_ = 106 ms, total recording time 2.5 h, processing with 40 Hz line broadening in both dimensions. (b) Short-delay ^1^H,^19^F correlation experiment (Tan et al. 2024). Parameters used: *t*_1max_ = 6.9 ms, *t*_2max_ = 86 ms, Δ_1_ = 7 ms, Δ_2_ = 2.5 ms, total recording time 8 h. The cross-peaks of residue 780 have the opposite sign because the chemical shift degeneracy prevents evolution under the ^2^*J*_FF_ coupling during the refocussing INEPT period of the experiment

### 2D NMR of R3H-F2

The 2D ^19^F-^19^F DQF-COSY spectrum shows that the diFIle residues in positions 761 and 733 display ^19^F chemical shifts differing by 3.3 and 5.1 ppm, respectively, whereas the ^19^F chemical shifts of the CHF_2_ group of residue 780 are degenerate (Fig. 4a). The short-delay ^1^H,^19^F correlation experiment shows the ^1^H-NMR signals of the C^δ^HF_2_ groups at about 5.7 ppm and of the C^γ1^H_2_ groups between 1.5 and 2.5 ppm (Fig. 4b). As in the case of R3H-F, the relative ^1^H chemical shifts observed in the wild-type protein are reiterated in R3H-F2 and we based the ^19^F NMR resonance assignments on this. The C^δ^HF_2_ groups of residues 761 and 733 show different cross-peak intensities with the different γ_1_-protons, indicating that the ^3^*J*_HF_ couplings are not averaged by rotation of the CHF_2_ groups. Instead, they preferentially populate conformations that produce large ^3^*J*_HF_ couplings for some of the ^19^F spins and small ^3^*J*_HF_ couplings for others.

## Discussion

To the best of our knowledge, lysozyme from bacteriophage λ produced with L-difluoromethionine (DFM) is to date the only example of a protein containing an aliphatic amino acid with a CHF_2_ group, where ^19^F-NMR spectra have been reported (Vaughan et al. 1999). The amino acid sequence of this protein contains three methionine residues. The ^19^F-NMR spectrum showed a spectral range of 5 ppm with degenerate chemical shifts for the two solvent-exposed DFM residues and an AB system for the buried residue 14. No comment was made regarding the stability of the protein preparation.

Our data indicate that both FIle and diFIle are recognized by the *E. coli* isoleucyl-tRNA synthetase. This allows uniform substitution of canonical isoleucine by fluorinated analogues but the strong preference for canonical isoleucine makes it difficult to avoid chemical heterogeneity arising from trace amounts of isoleucine.

Heterogeneity can also arise from chemical exchange between different conformations. Conformational exchange was documented by exchange cross-peaks for two of the FIle residues in the R3H-F sample. It is not clear, whether the same chemical exchange also prevails in R3H-F2. A NOESY spectrum recorded in 1.3 h with 60 ms mixing time showed no exchange cross-peak but the signal-to-noise ratio was low. The wild-type protein showed no evidence of slow conformational change.

Like the ^19^F-NMR spectra of proteins containing fluorinated leucine and valine residues, the ^19^F-NMR spectra of R3H-F and R3H-F2 display large spectral ranges, which invites the use of FIle and diFIle residues as site-specific probes that can be monitored by 1D NMR spectroscopy. Unfortunately, however, both FIle and diFIle caused rapid and irreversible sample degradation, with R3H-F2 precipitating faster than R3H-F. Recent results by Streit et al. (2024) indicate that the common observation of freshly produced protein being both soluble and folded can be attributed to the ribosome promoting the formation of the native structure of single-domain proteins even when destabilizing mutations prevent the refolding of the free protein after translation.

In the case of the R3H domain, the rapid sample degradation curtailed the time available to record 2D spectra for resonance assignment. We therefore based the assignment of the ^19^F-NMR spectra on the relative chemical shifts of the γ_1_-protons neighbouring the CH_2_F and CHF_2_ groups, which appear to be conserved as in the wild-type protein. We tested the validity of this assignment strategy by applying it to the ^19^F-resonance assignments of fluoroleucine, difluoroleucine, fluorovaline and difluorovaline residues incorporated into GB1, for which the assignments had been established by coupling and NOE connectivities to the protein backbone. In all cases, fully correct ^19^F assignments would have been obtained simply by relying on the conservation of ^1^H chemical shifts of the groups next to the CH_2_F groups as described in the present work. In the case of the R3H-F and R3H-F2 domains, the short-delay ^1^H,^19^F correlation experiment was sufficiently sensitive to yield the ^1^H chemical shifts of the γ_1_-protons with acceptable measurement times. This assignment strategy is more convenient than strategies relying on site-directed mutagenesis.

As Ile733 and Ile761 are near each other in the wild-type protein (supported by an NOE between their γ_2_-methyl groups), we anticipated the possibility of observing of a through-space ^19^F−^19^F coupling between the FIle residues at these sites. The AlphaFold model P38935 reports the same χ_1_ and χ_2_ rotamers as the NMR structure 1MSZ (Liepinsh et al. 2003), but with a somewhat greater distance between the δ-carbons of Ile733 and Ile761 (5.0 Å versus 3.8 − 4.6 Å). The apparent absence of a ^TS^*J*_FF_ coupling suggests that the AlphaFold model may be a more accurate structural representation at this location in the protein.

## Conclusions

Producing proteins with FIle in *E. coli* expression systems is difficult due to base-catalysed degradation of the amino acid that releases fluoride from the side chain. This problem will have to be overcome before the amino acid becomes attractive as a probe for monitoring by ^19^F-NMR spectra. The difluorinated analogue diFIle is chemically much more stable and it delivered a larger ^19^F spectral range, albeit at the expense of more complex ^19^F-NMR spectra and, in the case of the R3H domain, rapid protein precipitation. Given an effective assignment strategy that relies on a comparison of the ^1^H chemical shifts of the fluorinated residues with those of the canonical amino acid parents in the wild-type protein, fluorinated isoleucine analogues may yet open opportunities as easily detectable probes at isoleucine sites.

## Supplementary Information

Below is the link to the electronic supplementary material.

Supplementary Material 1

## Data Availability

Data are provided by the authors upon reasonable request.
