# Supplementary material for: 19F-NMR spectroscopy of fluorinated isoleucine analogues in a protein
Source: J Biomol NMR. 2026 Feb 17;80(1):7. doi: 10.1007/s10858-026-00488-z (PMC12913250; doi:10.1007/s10858-026-00488-z)
Supplement: Supplementary file 1 — Supplementary Material 1 [file 10858_2026_488_MOESM1_ESM.docx]

**Supporting Information**

**^19^F-NMR spectroscopy of fluorinated isoleucine analogues in a protein**

Adarshi P. Welegedara,^1^ Yi Jiun Tan,^1^ Matteo Borgini,^2,4^ Peter Wipf,^2,3^ Gottfried Otting^1^

^1^ ARC Centre of Excellence for Innovations in Peptide & Protein Science, Research School of Chemistry, Australian National University, Canberra, ACT 2601, Australia

^2^ University of Pittsburgh, Department of Chemistry, Pittsburgh, PA, 15260, USA

^3^ School of Pharmacy, University of Eastern Finland, Kuopio, 70210, Finland

^4^ Current address: Department of Chemistry and Biochemistry, Augusta University, Augusta, GA, 30912, USA

Email: [Gottfried.otting@anu.edu.au](mailto:Gottfried.otting@anu.edu.au) (ORCID 0000-0002-0563-0146)

**Table of contents**

**Detailed protocol of sample preparation for R3H-F and R3H-F2**

**Intact protein mass spectrometry**

**Figure S1.** 1D ^19^F-NMR spectra of the diFIle^.^HCl salt used for the cell-free protein synthesis of the R3H-F2 domain

**Figure S2.** Intact protein mass spectra of wild-type R3H domain and R3H-F2

**Figure S3.** [^19^F,^19^F]-NOESY spectrum of R3H-F

**Table S1.** DNA and corresponding amino acid sequence of the R3H domain of Sμbp-2 used

**References**

**Production of R3H-F2 and R3H-F**

The R3H domain of Sµbp-2 was produced with an N-terminal His_6_ tag followed by a TEV cleavage site. The gene was cloned between the *Nde*I and *Eco*RI sites of the T7 expression vector pETMCSI (Neylon et al., 2000). The protein sample with diFIle was produced by continuous exchange cell-free protein synthesis (CFPS) following an established protocol (Apponyi et al., 2008; Ozawa et al., 2012) with modifications to incorporate FIle or diFIle. The gene of Sµbp-2 R3H was amplified by PCR with eight-nucleotide single-stranded overhangs to generate DNA suitable for use in CFPS (Wu et al., 2007). Isoleucine was omitted when preparing the base-soluble fraction of the amino acid mixture. Every other amino acid was provided at 1 mM concentration.

In the case of R3H-F2, only 5 mg diFIle amino acid was available. All diFIle was dissolved in H_2_O and added to the outer buffer, yielding a final concentration of 0.35 mM. The pH of the outer buffer was adjusted to 7.5. The inner reaction mixture containing the S30 cell extract from *E. coli* BL21 Star (DE3) cells had a volume of 1 mL. The outer buffer had a volume of 10 mL. CFPS was conducted at 30 °C for 16 h.

In the case of R3H-F, the protocol of CFPS reaction had to be adjusted to account for the short lifetime of FIle in the reaction mixture. The adjustments included a shorter expression time (6 h instead of 16 h) and a higher concentration of FIle^.^HCl (2 mM final concentration, 0.1 mL added every hour from a 100 mM aqueous stock solution, each addition accompanied by pH adjustment of the outer buffer to pH 7 to 7.5 if needed). As the CFPS conducted with 1 mL inner reaction mixture yielded only 0.1 mg protein after purification, the reaction was conducted in 40 separate batches and the inner reaction mixtures were combined before protein purification.

To purify the proteins, the inner reaction mixture was first centrifuged at 18000 *g* for 30 minutes at 4 ^o^C. Next, the proteins were loaded onto a 1 mL Ni-NTA gravity column (GE Healthcare) equilibrated with buffer A (50 mM Tris−HCl, pH 7.5, 300 mM NaCl) and washed with buffer B (the same as buffer A but with 10 mM imidazole). The proteins were eluted with buffer C (the same as buffer A but with 300 mM imidazole). The samples were concentrated and exchanged into NMR buffer (10 mM sodium phosphate and 100 mM NaCl, pH 6.3) by ultrafiltration using an Amicon (Millipore) centrifugal filter device with a 3‐kDa molecular mass cutoff. The concentration of the protein was determined using a BCA (bicinchoninic acid) protein assay kit (Thermo Scientific). 10% D_2_O were added prior to NMR measurements.

**Intact protein mass spectrometry**

Intact protein analysis was conducted using a Thermo Fisher Scientific UltiMate 3000 HPLC system connected to an Orbitrap Fusion™ Tribrid™ mass spectrometer (Thermo Fisher Scientific, USA). The HPLC system is connected to a ZORBAX 300SB-C3 column (3.5 µm, 4.6 x 50 mm; Agilent Technologies, USA). Approximately 30 pmol of the protein sample was injected and separated using a 500 µL/min linear gradient of 0.1% formic acid in water (solvent A) and 0.1% formic acid in acetonitrile (solvent B), with solvent B increasing from 5% to 80% over 7 min. Data was acquired in positive ion mode using an electrospray ionization (ESI) source. The intact protein mass was determined by deconvolution using Xcalibur 3.0.63 software (Thermo Fisher Scientific, USA).


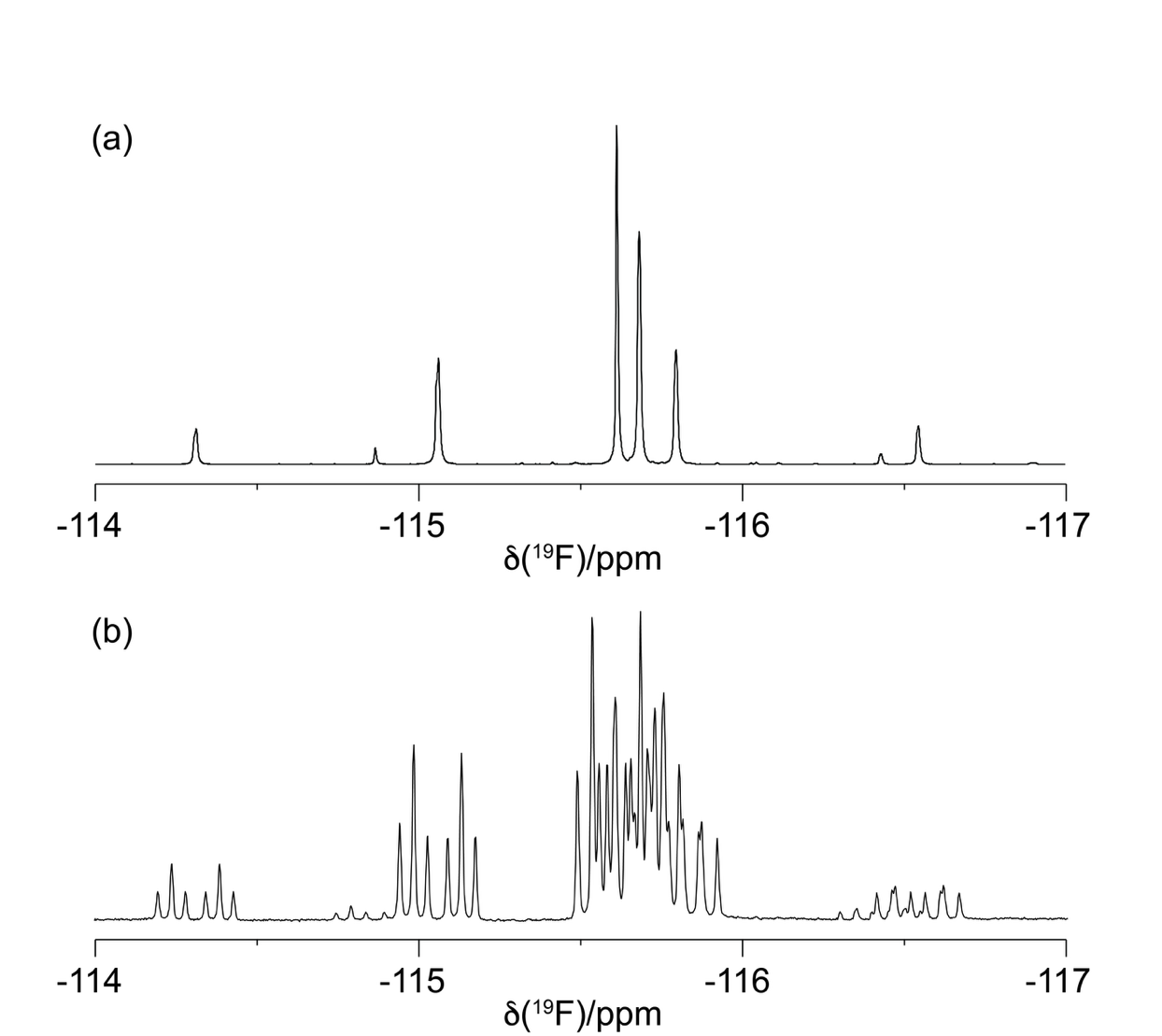


**Figure S1.** 1D ^19^F-NMR spectra of the diFIle^.^HCl salt used for the cell-free protein synthesis of the R3H-F2 domain. The NMR spectra were recorded on a 400 MHz NMR spectrometer in aqueous solution at 25 ^o^C. (a) Spectrum recorded with ^1^H broadband decoupling. It shows two overlapping AB spectra of integral ratio 3:2, with chemical shifts of -115.2 and -116.1 ppm for the major species, and -114.7 and -116.2 ppm for the minor species. The ^2^*J*_FF_ coupling constant is 282 Hz. (b) Spectrum recorded without decoupling, showing doublets of triplets with ^2^*J*_FH_ = 56 Hz and ^3^*J*_FH_ = ~18 Hz.

**
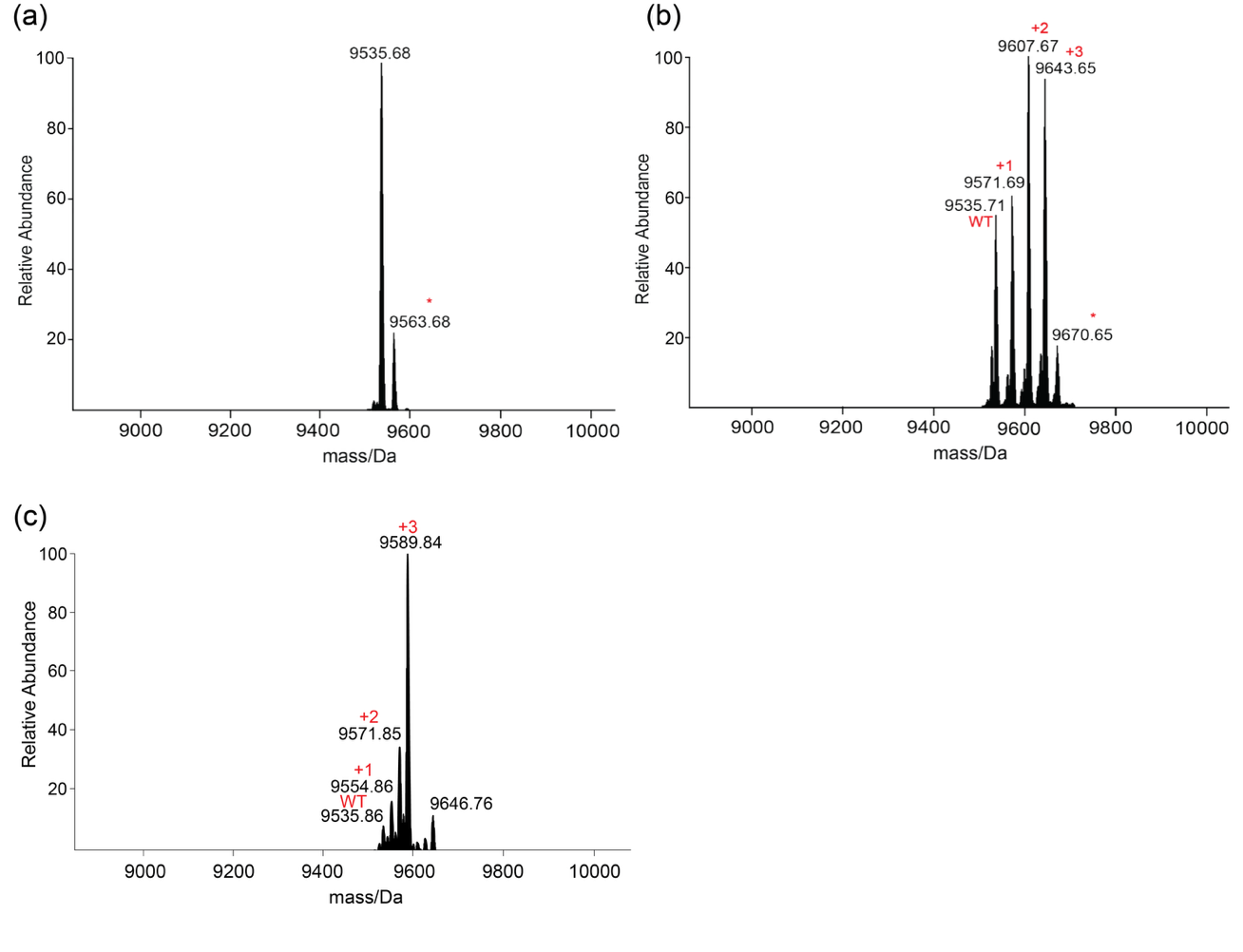
**

**Figure S2.** Intact protein mass spectra of wild-type R3H domain and R3H-F2. The mass labelled with a star is of protein including a formyl group at the amino terminus. (a) Wild-type R3H domain. The calculated mass is 9536.73 and 9564.72 Da with the N-formyl group. (b) R3H-F2. The species containing only two diFIle residues (labelled +2) was slightly more abundant than the species with all canonical isoleucine residues replaced by diFIle (labelled +3). The calculated masses are 9572.71, 9608.69 and 9644.67 Da for the protein containing, respectively, one, two or three diFIle residues. The mass calculated for N-formylated protein with three diFIle residues is 9672.66 Da. (c) R3H-F. The calculated masses are 9554.70, 9572.70 and 9590.70 Da for the protein containing, respectively, one, two or three FIle residues.

**
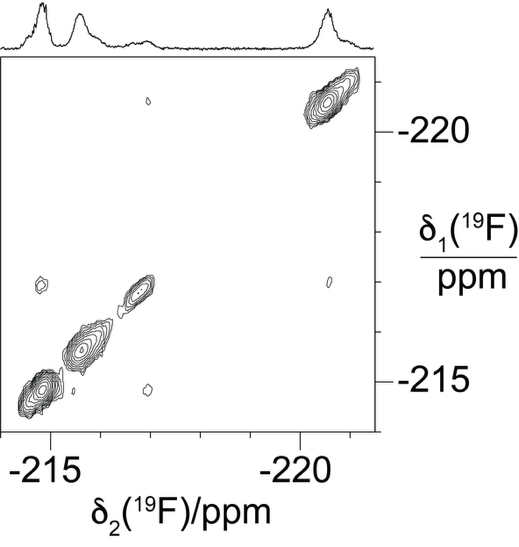
**

**Figure S3.** [^19^F,^19^F]-NOESY spectrum of R3H-F recorded without ^1^H decoupling. Parameters used: 150 ms mixing time, *t*_1max_ = 3.6 ms, *t*_2max_ = 102 ms, total recording time 19 h.

**Table S1.** DNA and corresponding amino acid sequence of the R3H domain of Sμbp-2 used in the current work.

| Protein | DNA sequence | Amino acid sequence |
| --- | --- | --- |
| His_6_-TEV-R3H | ATGCATCATCATCACCATCACGAAAACCTGTATTTTCAGGGTGTTGAAAGCCAGGATGGTGTTGATCATTTTCGTGCAATGATCGTTGAATTTATGGCCAGCAAAAAGATGCAGCTGGAATTTCCGCCTAGCCTGAATAGCCATGATCGTCTGCGTGTTCATCAGATTGCCGAAGAACATGGTCTGCGTCATGATAGCAGCGGTGAAGGTAAACGTCGTTTTATTACCGTTAGCAAACGTGCC | MHHHHHHENLYFQGVESQDGVDHFRAMIVEFMASKKMQLEFPPSLNSHDRLRVHQIAEEHGLRHDSSGEGKRRFITVSKRA |

**References**

Apponyi MA, Ozawa K, Dixon NE, Otting G (2008) Cell-free protein synthesis for analysis by NMR spectroscopy. In *Structural Proteomics*, Methods in Molecular Biology; Springer; Vol. 426, pp 257−268

Neylon C, Brown SE, Kralicek AV, Miles CS, Love CA, Dixon NE (2000) Interaction of the *Escherichia coli* replication terminator protein (Tus) with DNA: a model derived from DNA- binding studies of mutant proteins by surface plasmon resonance. Biochemistry 39:11989−11999

Ozawa K, Loscha KV, Kuppan KV, Loh CT, Dixon NE, Otting G (2012) High-yield cell-free protein synthesis for site-specific incorporation of unnatural amino acids at two sites. Biochem Biophys Res Commun 418:652−656

Wu PSC, Ozawa K, Lim SP, Vasudevan S, Dixon NE, Otting G (2007) Cell-free transcription/translation from PCR amplified DNA for high-throughput NMR studies. Angew Chem Int Ed 46:3356−3358
